# PBN11-8, a Cytotoxic Polypeptide Purified from Marine *Bacillus*, Suppresses Invasion and Migration of Human Hepatocellular Carcinoma Cells by Targeting Focal Adhesion Kinase Pathways

**DOI:** 10.3390/polym10091043

**Published:** 2018-09-19

**Authors:** Lanhong Zheng, Xiangjie Zhu, Kangli Yang, Meihong Zhu, Ammad Ahmad Farooqi, Daole Kang, Mi Sun, Yixin Xu, Xiukun Lin, Yingang Feng, Fangfang Liang, Fuming Zhang, Robert J. Linhardt

**Affiliations:** 1 School of Pharmacy, Shanghai University of Medicine & Health Sciences, Shanghai 201318, China; zhenglanhong@126.com (L.Z.); xuyx@sumhs.edu.cn (Y.X.); 2 Key Laboratory of Sustainable Development of Polar Fishery, Ministry of Agriculture, Yellow Sea Fisheries Research Institute, Chinese Academy of Fishery Sciences, Qingdao 266071, China; zhuxiangjie1204@163.com (X.Z.); hnysykl@163.com (K.Y.); zhumhlv@163.com (M.Z.); kdlqust@126.com (D.K.); sunmi@ysfri.ac.cn (M.S.); liangfangfang12@163.com (F.L.); 3 Shanghai Ocean University, Shanghai 201306, China; 4 Institute of Biomedical and Genetic Engineering (IBGE), Islamabad 44000, Pakistan; ammadfarooqi@rlmclahore.com; 5 Department of Pharmacology, Southwest Medical University, Luzhou 646000, Sichuan, China; 6 Shandong Provincial Key Laboratory of Energy Genetics and Qingdao Engineering Laboratory of Single Cell Oil, Institute of Bioenergy and Bioprocess Technology, Chinese Academy of Sciences, 189 Songling Road, Qingdao 266101, China; fengyg@qibebt.ac.cn; 7 Departments of Chemistry and Chemical Biology, Chemical and Biological Engineering, Biology and Biomedical Engineering, Center for Biotechnology and Interdisciplinary Studies, Rensselaer Polytechnic Institute, Troy, NY 12180, USA; zhangf2@rpi.edu (F.Z.); linhar@rpi.edu (R.J.L.)

**Keywords:** polypeptide, cytotoxicity, FAK, invasion

## Abstract

The development of antitumor drugs has attracted cancer researchers and the identification of novel antitumor lead compounds is certainly of great interest. The fermentation broth of *Bacillus* sp. N11-8, which was isolated from the Antarctic waters, showed cytotoxicity towards different cells. A cytotoxic polypeptide, PBN11-8, was purified from the fermentation broth of *Bacillus* sp. N11-8 using ultrafiltration, ammonium sulfate precipitation, anion exchange liquid chromatography and high performance liquid chromatography (HPLC). Cloning and sequence analysis showed that PBN11-8 polypeptide (MW: ~19 kDa by the electrospray-ionization (ESI)) displayed high similarity with peptidase M84 from *Bacillus pumilus*. PBN11-8 possessed moderate cytotoxicity towards several cancer cell lines with IC_50_ values of 1.56, 1.80, 1.57, and 1.73 µg/mL against human hepatocellular carcinoma cell line BEL-7402, human renal clear cell adenocarcinoma cell line 786-0, human hepatocellular carcinoma cell line HepG2, and human pancreatic cancer cell line Panc-28, respectively. Moreover, the polypeptide displayed weak cytotoxicity towards normal cell line renal tubular epithelial cell line HK2 and human normal liver cell line L02 cells. Wound healing migration and Transwell experiments demonstrate that PBN11-8 could inhibit the migration and invasion of BEL-7402. Further investigation revealed that PBN11-8 suppresses focal adhesion kinase (FAK)-mediated adhesion, migration, and invasion by disturbing FAK/extracellular regulated protein kinases (ERK) signaling and matrix metalloproteinase-2(MMP-2) and matrix metalloproteinase-9 (MMP-9) in BEL-7402 cells. Thus, PBN11-8 represents a potential novel anti-cancer lead compound.

## 1. Introduction

The worldwide incidence of cancer increases year by year due to environmental pollution and aging. Thus, it is of great significance to discover and study new anti-tumor drugs. The oceans, accounting for about three-quarters of the earth’s surface, contain extremely diverse resources [1], including aquatic species and various microbes. Marine microbes are an important source of new drugs and functional foods. These microbes produce large numbers of unusual natural compounds with unique structures and physiological activities required for their growth and metabolism in an extremely adverse living environment (i.e., high salt, high pressure, lack of oxygen, lack of sunlight). The most dominant marine microbes, marine bacteria, produce a wide variety of novel bioactive substances and have become an important resource in new drug screening.

The metabolites of marine bacteria with a variety of bio-functions, such as antitumor activity, have been isolated from marine sediment, seawater, algae, and the surface of marine animals [2]. Didemnin B originating from Caribbean tunicate *Trididemnum solidum* was the first marine antitumor drug to enter clinical studies. Recent studies have shown that didemnin B is produced by didemnin’s biosynthetic gene clusters of the marine alpha-deformation bacteria, *Tistrellamobilis* and *Tistrellabauzanensis* [3]. A novel bioactive peptide, SBP, was isolated through the fermentation of the marine *Brevibacillus* sp. S-1 by our research group, and it demonstrates wide antitumor activity [4]. An extracellular l-asparaginase is produced by a protease-deficient isolate, *Bacillus aryabhattai* ITBHU02, and *in vitro* cytotoxicity assays with HL60 and MOLT-4 cell lines indicate that the enzyme has significant antineoplastic properties [5].

The integrin family of receptors are key ligands of cell adhesion to the extracellular matrix (ECM), and these receptors provide the links of ECM to the actin cytoskeleton [6,7,8]. Focal adhesion kinase (FAK), the first identified receptor, is a vital signaling molecule for cell motility and invasion. Integrin/FAK signaling has been reported to activate many signaling pathways for promoting tumorigenesis [9,10,11,12]. In the present research, we isolated a polypeptide, PBN11-8, from a marine bacterium *Bacillus* sp. N11-8, which displayed high antitumor activity against several cancer cell lines. PBN11-8 can affect the migration and invasion of BEL-7402 cells, as analyzed using the Scratch-wound assay and the Transwell experiment. Further study revealed that the polypeptide PBN11-8 is able to disturb the FAK signaling, and abrogate cancer cell motility and invasiveness and antitumor protein may be a novel polypeptide for targeting FAK signaling.

## 2. Materials and Methods

### 2.1. Cell Culture

Human hepatocellular carcinoma cell line (BEL-7402 and HepG2), human pancreatic cancer cell line (Panc-28), and renal tubular epithelial cell line (HK2) were provided by the Institute of Marine Science, Chinese Academy of Sciences, Qingdao, China. Human renal clear cell adenocarcinoma cell line (786-0) and human normal liver cell line (L-02) were obtained from the Chinese Academy of Sciences Typical Culture Collection Commission Cell library/Chinese Academy of Sciences, Shanghai Institute of Life Science, Cell Resource Center, Shanghai, China. All cell lines were grown in the recommended media supplemented with 10% FBS and cultured at 37 °C in a humidified atmosphere of 5% CO_2_.

### 2.2. Materials

Hiprep Q FF 16/10 column was purchased from GE Healthcare (Uppsala, Sweden). Protein-PAKTM60 was purchased from Waters (Milford, MA, USA). Penicillin-steptomycin, 3-(4,5-dimethylthiazol-2-yl)-2,5-diphenyltetrazolium bromide (MTT), dimethyl sulfoxide (DMSO) were purchased from sigma (St. Louis, MO, USA), Dulbecco’s Modified Eagle’s Medium (DMEM) and fetal calf serum were products of Gibco Invitrogen (Carlsbad, CA, USA). BCA (Bicinchoninic acid) protein assay kit was purchased from Thermo Scientific (Pierce Inc., Rockford, IL, USA). Integrin β1, FAK, p-FAK, AKT, p-AKT, ERK, p-ERK, MMP-2, MMP-9, GAPD Hantibodies were obtained from Cell Signaling technology (Beverly, MA, USA). Peroxidase-Conjugated Affiniure Goat Anti-Rabbit IgG (H + L) secondary antibodies were purchased from Origene (Rockville, FL, USA).

### 2.3. Microorganism and Fermentation

*Bacillus* sp. N11-8 was isolated from the Antarctic surface seawater [13]. The basal medium consisted of 1.0% tryptone, 0.3% beef extract, and 0.5% NaCl (pH 6.5–7.0). Incubation was carried out at 25–30 °C for 60–72 h in a rotary shaker.

### 2.4. Purification and Identification of Polypeptide

After the fermentation broth centrifugation, the supernatant was separated by an ultrafiltration membrane of molecular-weight 3 and 30 kDa cut-off, and antitumor activity was detected by the MTT method, and the active component was selected for further purification [14]. Crystals of ammonium sulfate were added to the supernatant to give 50% and 75% saturation. The solution was stored for 3 h before precipitation. The resulting precipitates were recovered by centrifugation, which was dissolved in Tris-HCl buffer (20 mM, pH 7.0), and dialyzed against the same buffer solution [15]. Precipitates formed during dialysis were removed by centrifugation. The fraction that had the strongest cytotoxicity activity was used for further experiments.

The active fraction was dissolved in Tris-HCl buffer (50 mM, pH 7.96) and loaded onto a HiPrep Q FF 16/10 column (Uppsala, Sweden), which had been previously been equilibrated with the above buffer [16]. The adsorbed proteins were eluted with 0–100% 1 mol/L NaCl in the same buffer at a flow rate of 3 mL/min. Each fraction monitored at 280 nm was collected [17]. All the fractions were desalted against ultra-pure water (dialyzing) and the antitumor activities were determined. The fraction with the highest activity was used in subsequent experiments.

HPLC was performed on a Waters 2545-2767-2489 HPLC system (Milford, MA, USA) fitted with a Waters Protein-PAKTM 60 column (Milford, MA, USA). The elution solvent system was composed of water-ammonium acetate (solvent A, 100:0.1, *v*/*v*) and methanol-ammonium acetate (Solvent B, 100:0.1, *v*/*v*). The activity fraction was further purified using a gradient elution 0–5% of solvent B in 10 min, then 100% of solvent A in 5 min at a flow rate of 3 mL/min. Notably, all of the eluted samples were monitored using a UV-Spectrophotometer (Waters, Milford, MA, USA) at wavelength 280 nm. Protein concentrations were determined using the BCA protein assay kit with bovine serum albumin (BSA) as the standard.

Purity was determined by 12% sodium dodecyl sulfate-polyacrylamide gel electrophoresis (SDS-PAGE) and analytical type HPLC. The active fractions were collected and analyzed by SDS-PAGE. The gels were stained for 30 min with Coomassie blue R-250 (Solarbio, Beijing, China) and strained for 3 h with 20% methanol [18].

Molecular Mass was determined with a Bruker microTOF accurate mass instrument (Billerica, MA, USA), equipped with an electrospray-ionization (ESI) source operated in the positive mode. The main source setting data were as follows: capillary voltage, 4500 V; nebulizer pressure, 2.4 bar; drying gas flow (N_2_), 8 L·min^−1^; and drying temperature, 210 °C. Mass spectra were collected between *m*/*z* 200 and *m*/*z* 500. The sample was dissolved with deionized H_2_O [19].

The *N*-terminal amino acid sequence was analyzed using the Edman degradation approach [20]. Briefly, proteins were resolved using SDS-PAGE, transferred onto sequencing grade Polyvinylidene fluoride (PVDF, 0.2 mm, Bio-Rad, Richmond, CA, USA), and stained for 1 min with 0.1% Coomassie blue R-250 in 50% methanol. After washing with 50% methanol and H_2_O, the sample was air-dried. The *N*-terminal sequence was analyzed with Precise 491 sequencing system (Applies Biosystems, Foster City, CA, USA).

### 2.5. Cloning, Sequence Analysis of PBN11-8

The primers were designed based on the *N*-terminal sequence, and the full-length of gene of PBN11-8 was amplified using Polymerase Chain Reaction (PCR) technology. Homology analysis was performed using Blast search (www.ncbi.nih.gov) on the basis of the whole gene sequence of PBN11-8.

### 2.6. Measurement of Protease Activity

Folin-Phenol Reagent Method was used to measure the protease activity of PBN11-8 using casein as substrate. The reaction was reacted for 10 min at 37 °C in a reaction volume of 1 mL substrate in Tris-HCl buffer (50 mM, pH 7.96) and 1 mL of enzyme solution. After adding 2 mL of 0.4 M trichloroacetic acid (TCA), the mixture was allowed to stand for 10 min. The mixture was then centrifuged to remove the precipitation. Supernatant (1 mL) was mixed with 5 mL Na_2_CO_3_ (0.4 M) and 1 mL Folin-phenol reagent. The mixture was left at 37 °C for 20 min and the Optical Density (OD) value was measured at 680 nm. The blank experiment was reacted in the same manner, except that the enzyme was added after the addition of TCA. One unit (U) of protease activity was defined as generating the amount of 1 μg tyrosine with per gram enzyme in one minute.

### 2.7. Cytotoxic Activity Assay

MTT assay was used to assess cytotoxicity of PBN11-8 [21]. About 4000 cells were seeded into each well on 96-well microplates and cultured overnight. Then, the purified polypeptide PBN11-8 was added at certain concentrations into the wells. After incubation for 48 h, 20 µL of MTT (0.5 mg/mL) was added to each well and incubated for an additional 4 h. Then, the media were removed and 150 µL DMSO was added. The absorbance of samples was evaluated at 570 nm with an Infinite M200 PRO microplate reader (TECAN Group Ltd., Mannerdorf, Switzerland). The cytotoxicity of the PBN11-8 was expressed as an IC_50_ value, defined as the concentration causing a 50% reduction of cell viability compared with untreated cells. The percentage of cytotoxicity was calculated as follows: Relative inhibition rate (%) = [(*A*570 value of the control − *A*570 value of the experimental samples)/*A*570 value of the control] ×100% [21].

### 2.8. Crystal Violet Adhesion Assay

BEL-7402 cells were seeded in 12-well microplates and cultured overnight. PBN11-8 was then added to a final concentration of 2 µg/mL. After incubation for 0, 12, 24, and 48 h, the supernatant was removed and the cells were washed three times with PBS, and then the cells were treated with 10% methanol for 30 s. Crystal violet solution (0.5 mL of 2%) was added in each well and incubated it at room temperature for 20 min. Thereafter, the media was removed and the cells were washed with distilled water. Finally, the cells were observed under a microscope. To value the concentration dependent manner, the crystal violet adhesion assay was performed on BEL-7402 cells treated for 12 h with (or without) treatment of 1, 2, and 4 µg/mL of PBN11-8.

### 2.9. Migration and Invasion Assay on BEL-7402 Cells

The migration ability of BEL-7402 cells was assessed by Scratch-wound assay and Transwell assays. In the cell migration assay, BEL-7402 cells were treated with PBS or with 0.5 μg/mL polypeptide PBN11-8. After incubation for 12 and 24 h, cell migration was analyzed using a Scratch-wound assay. For cell invasion assay, BEL-7402 cells were treated without or with 0.25, 0.5, or 1 μg/mL PBN11-8 polypeptide separately. After being cultured for 24 h, non-invading cells on the upper surface were removed, and the invasive cells on the lower surface were stained with 0.1% crystal violet. The stained invasive cells were observed using an inverted light microscope. The invasive cell numbers (as the mean from six random fields) were counted manually under the microscope. Results in each group are normalized to those from PBS treated cells [22].

### 2.10. Immunoblotting Assay

Cell lysates were harvested with RIPA buffer, which contained a protease inhibitor cocktail (1 mM phenylmethanesulfonyl fluoride and 1 μg/mL leupeptin) and phosphatase inhibitor cocktail (1 mM sodium fluoride and 1 mM sodium orthovanadate). Equal quantities of cell extract were resolved by 12% SDS-PAGE. Proteins were transferred to PVDF membranes and blocked with 5% fat-free skim milk for 1.5 h, and incubated with primary antibodies at 4 °C overnight. After that, the membranes were washed with TBST and incubated with a horseradish peroxidase-conjugated secondary antibody for 2 h. Protein bands were visualized by enhanced chemiluminescence (Millipore, MA, USA) and analyzed by densitometry.

### 2.11. Quantitative Real-Time Reverse Transcription Polymerase Chain Reaction (qRT-PCR) Assay

The relative mRNA levels of integrin β1, FAK in the BEL-7402 cells of each group was respectively determined by qRT-PCR assay. qRT-PCR was performed by a ABI StepOne Real-time PCR system (Applied Biosystems, foster, CA, USA). The total reactive volume is 20 μL, including 10 μL of 2 × SYBR Premix Ex TaqⅡ, 0.4 μL of 50 × ROX Reference Dye (TaKaRa, Dalian, China), 0.8 μL of 10 μM forward primer, 0.8 μL of 10 μΜ reverse primer, 2 μL of cDNA, double distilled water 6 μL. The reaction was performed as the manufacture’s instruction: incubation at 95 °C for 30 s followed by 40 cycles of 95 °C for 5 s and 60 °C for 30 s. Total RNA was extracted using Trizol Plus RNA purification kit (Tiangen, Beijing, China). Concentration of RNA was determined by absorption at 260 and 280 nm. The quantitative analysis was conducted by using the formula as follows: Δ*C*t = *C*t1 − *C*t2, where the *C*t1 was *C*t value of target gene and *C*t2 was *C*t value of GAPDH. The results were presented in 2^−ΔΔ*C*t^. The experiment was repeated three times (*n* = 3). The sequences of primers were as follows:

GAPDH forward: 5′-AAGTTCAACGGCACAGTCAAGG-3′,
GAPDH reverse: 5′-CATACTCAGCACCAGCATCACC-3′;
Integrin β1forward: 5′-TTCGATGCCATCATGCAAGTTG-3′,
Integrin β1 reverse: 5′-CCATCTCCAGCAAAGTGAAACC-3′,
FAK-forward: 5′-ACTCATCGAGAGATCGAGATGG-3′,
FAK reverse: 5′-GCCCTAGCATTTTCAGTCTTGC-3′.

### 2.12. Statistical Analysis

The experiments were conducted at least three times and the experimental data were expressed as the mean ± SD. The statistical significance of the mean difference between the control and treated groups was determined by a paired *t*-test; * *p* < 0.05 vs. control; and ** *p* < 0.01 vs. control.

## 3. Results

### 3.1. Preparation of Crude Extract

Approximately 50 L of production media was prepared to screen the cytotoxic components. Membranes which had different pore sizes were selected to identify the main fractions with the cytotoxicity. The fraction of 3–30 kDa had the highest cytotoxicity, and thus, it was chosen for subsequent experiments. The fraction of 3–30 kDa was then treated by ammonium sulfate precipitation. The supernatant fraction at 50% saturation was shown to exert the strongest cytotoxic activity.

### 3.2. Purification and Identification of Cytotoxic Polypeptide

**Figure 1:**
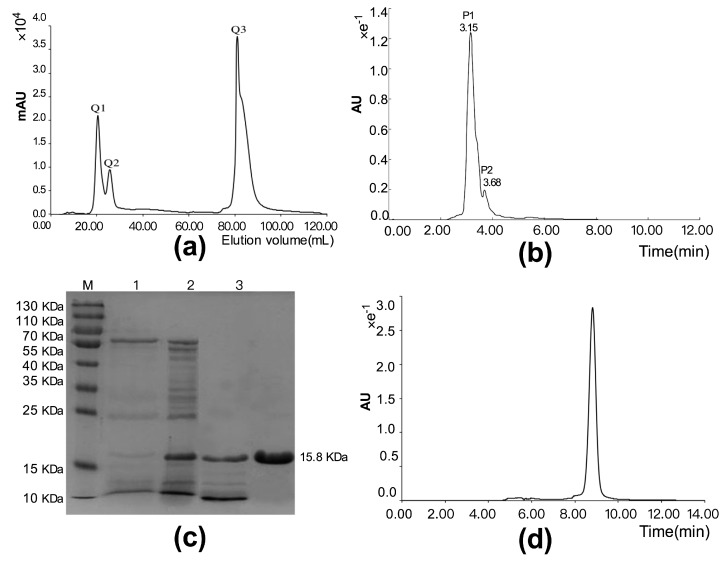

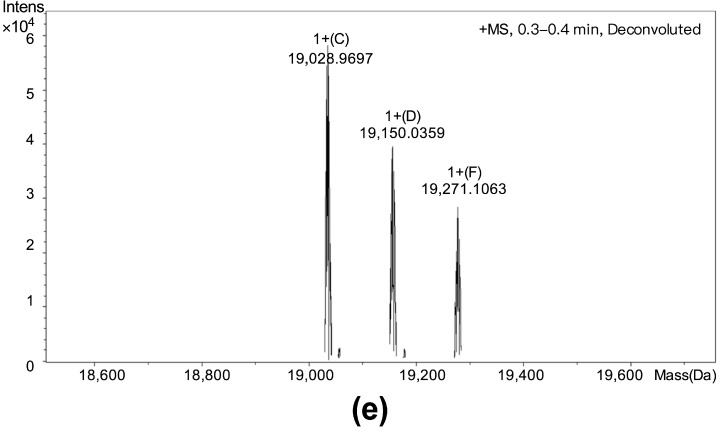
Purification and identification of polypeptide PBN11-8. (**a**) Purification of the crude polypeptide by strong anion-exchange chromatography on a HiPrep Q FF 16/10 column. (**b**) Purification of the Q chromatographic fraction Q2 by high performance liquid chromatography (HPLC) on a Protein-PAKTM 60 column. (**c**) Detection of polypeptide by 12% sodium dodecyl sulfate-polyacrylamide gel electrophoresis (SDS-PAGE). Lane M：MW Mark; Lane 1: total proteins; Lane 2: ammonium sulfate precipitation; Lane 3: strong anion exchange fraction; Lane 4: purified polypeptide. (**d**) Purity analysis of P1 by analytical Protein-PAKTM60 hydrophilic molecular exclusion chromatography. (**e**) The electrospray-ionization (ESI) mass spectrum of the P1.

We next developed a purification protocol involving strong anion-exchange chromatography and high performance liquid chromatography. The crude extract was dissolved with the Tris-HCl buffer (50 mM, pH 7.96), and the dissolved solution was applied to a Hiprep Q Fast Flow column. The results show the penetration peak fractions Q1 and Q2, and the elution peak fraction Q3 (Figure 1a). Fraction Q2 with the strongest cytotoxicity was subjected to high performance liquid chromatography using a Protein-PAKTM60 column, and two peak fractions, P1 and P2, were obtained (Figure 1b). The results of the cytotoxic activity evaluation suggested that fraction P1 had the stronger activity in suppressing the proliferation of BEL-7402 cells. We designated this cytotoxic polypeptide as PBN11-8. The peak P1 sample displayed a single band (Figure 1c) as analyzed by SDS-PAGE. The purity of P1 sample was also detected by analytical Protein-PAKTM60 hydrophilic molecular exclusion chromatography. The result confirmed peak P1 sample as a single peak (Figure 1d). The above data indicate that PBN11-8 polypeptide is highly purified. ESI-TOF analysis of PBN11-8 was performed with differentiation signals in the mass of about 19 kDa (Figure 1e), and the *N*-terminal partial sequence of PBN11-8 was determined to be ASTGSQKVTVYAVAD using Edman degradation.

### 3.3. Cloning, Sequence Analysis and the Protease Activity of Polypeptide PBN11-8

**Figure 2:**
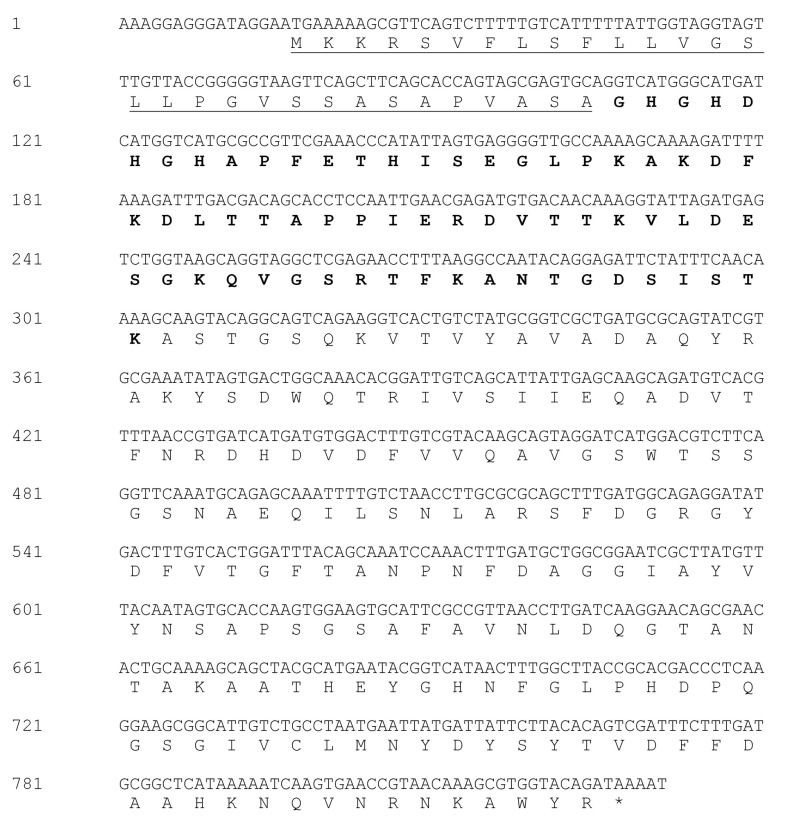
Nucleotide and amino acid sequence of PBN11-8. The full length of PBN11-8 cDNA was cloned using the degenerate primers approach. The amino acid sequence of PBN11-8 was deduced from the cDNA sequence. The asterisk indicates a stop codon. Signal peptide sequence is underlined, and the whole sequence of PBN11-8 amino acid residues is presented in bold.

The full length of PBN11-8 cDNA was cloned using degenerate primers approach. The amino acid sequence of PBN11-8 was deduced from the cDNA sequence (Figure 2). The signal peptide was identified using the SignalP 4.1 Server (http://www.cbs.dtu.dk/services/SignalP). The entire amino acid sequence of PBN11-8 was found to share 98.5% similarity with peptidase M84 of *Bacillus pumilus* (GenBank accession WP_025208148). These results confirmed that PBN11-8, similar to peptidase M84 of *Bacillus*, belonged to a type of zinc-dependent metalloprotease family, characterized by a consensus amino acid sequence HExxH, and the histidines are responsible for zinc ligands and the glutamic acid functions as a catalytic base. A third zinc ligand is provided by the side-chain of His, Glu, or Asp, usually located downstream of this motif [23]. Enzymatic analysis confirmed that PBN11-8 displayed 1.38 million U/g protease activity as measured using Forint phenol method.

### 3.4. PBN11-8 Displayed Cytotoxicity toward Different Cells

**Figure 3:**
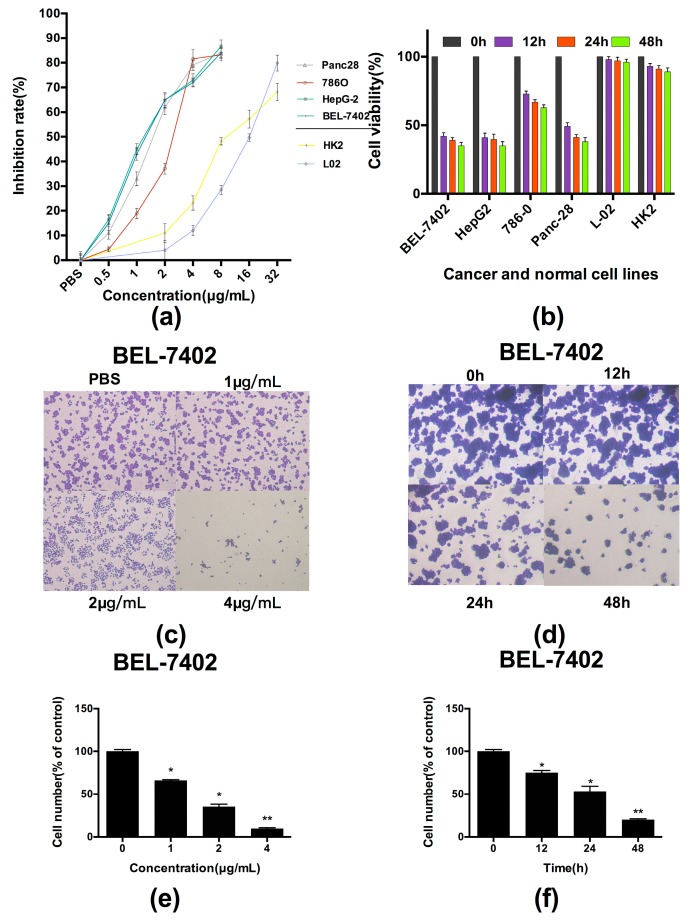
PBN11-8 displays potent cytotoxicity to cancer cells. (**a**) Cells were treated with certain concentrations of PBN11-8. The cell inhibitory rate was determined by MTT assay as described in the experimental section. The IC_50_ values were 1.56, 1.80, 1.57 and 1.73 µg/mL for BEL-7402, 786-0, HepG2 and Panc-28 cells, respectively. The IC_50_ values were 11.79 and 14.72 µg/mL for HK2 and L02 cells, respectively. (**b**) Cells were cultured in 96-well plate and treated with 4 µg/mL PBN11-8 for each cell lines for 0,12, 24, and 48 h to study the time dependent analysis. The cell viability was analyzed by MTT assay. (**c**) The results of the crystal violet adhesion assay in BEL-7402 cells induced by 1, 2, 4 µg/mL of PBN11-8 for 12 h. (**d**) The results of the crystal violet adhesion assay in BEL-7402 cells induced by 2 µg/mL of PBN11-8 for 12, 24 and 48 h. (**e**,**f**) The quantitative evaluations of the crystal violet adhesion assay. Data represent the mean ± SD of three independent experiments. * *p* < 0.05 vs. control; and ** *p* < 0.01 vs. control.

A panel of cancer cell lines was investigated using MTT assay to evaluate the inhibition of proliferation by PBN11-8. Figure 3a showed that PBN11-8 significantly inhibited the viability of the selected nine cancer cell lines in a dose-dependent manner. After treatment for 48 h, the IC_50_ values were 1.56, 1.80, 1.57, and 1.73 µg/mL for BEL-7402, 786-0, HepG2, and Panc-28 cells, respectively. Moreover, the polypeptide only displayed weak cytotoxic activity toward normal cells; the IC_50_ values were 11.79 and 14.72 µg/mL for HK2, and L02 cells, respectively. To evaluate the time-dependent inhibition, cancer and normal cell lines were exposed to 4 µg/mL of PBN11-8 for 12, 24, and 48 h. As shown in Figure 3b, treatment with PBN11-8 more obviously inhibited the growth of cancer cells than that in normal cells; the viability of cancer cells decreased to more than 40%, while the viability only decreased to less than 80% in normal cells.

Crystal violet adhesion assay indicated that PBN11-8 significantly weakened BEL-7402 cell adhesion and survivability in a dose-dependent manner (Figure 3c,e) and a time-dependent manner (Figure 3d,f).

### 3.5. PBN11-8 Affects the Migration and Invasion of BEL-7402 Cells

**Figure 4:**
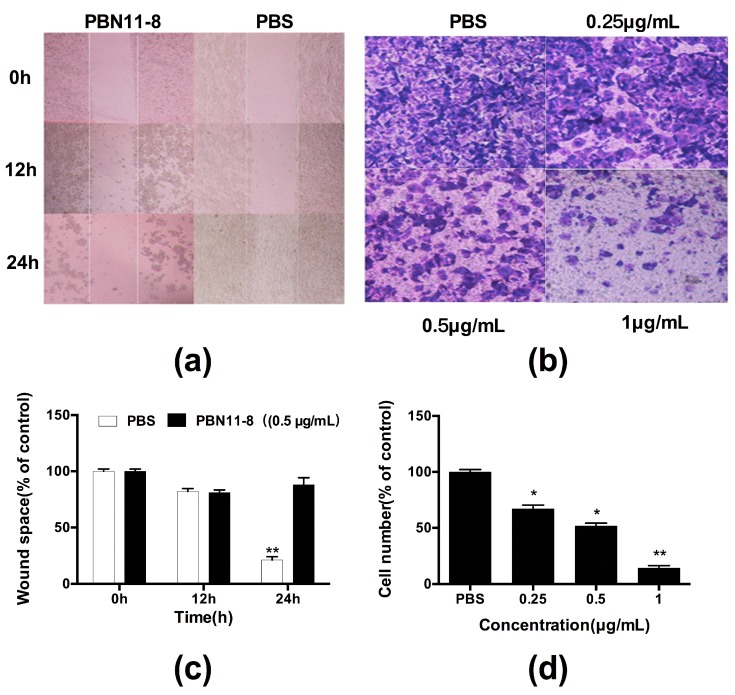
Effect of PBN11-8 on migration and invasion of BEL-7402 cells. (**a**) The scratch test results of the cell migration. (**b**) The detection results of the cell invasion. Quantitative evaluations of BEL-7402 migration induced by PBN11-8 in the Scratch-wound assay (**c**) and Transwell assay (**d**). Data represent the mean ± SD. of three independent experiments. * *p* < 0.05 vs. control; and ** *p* < 0.01 vs. control.

Both Scratch-wound assay and Transwell assay were employed to investigate the influence of PBN11-8 on BEL-7402 cells migration. The results of Scratch-wound assay clearly showed that 0.5 µg/mL PBN11-8 reduced the wound healing activity of BEL-7402 (Figure 4a,c). In the Transwell assay, the data revealed that PBN11-8 caused a concentration-dependent reduction in the number of migrating cells (Figure 4b). When cells were treated with (and without) 0.25, 0.5, and 1.0 µg/mL of PBN11-8, the invasion ability of the cancer cells was inhibited in a dose-dependent manner (Figure 4d). All the results indicated that PBN11-8 clearly inhibited the migration and invasion of BEL-7402 cells.

### 3.6. PBN11-8 Inhibited the Activation of FAK in BEL-7402 Cells

**Figure 5:**
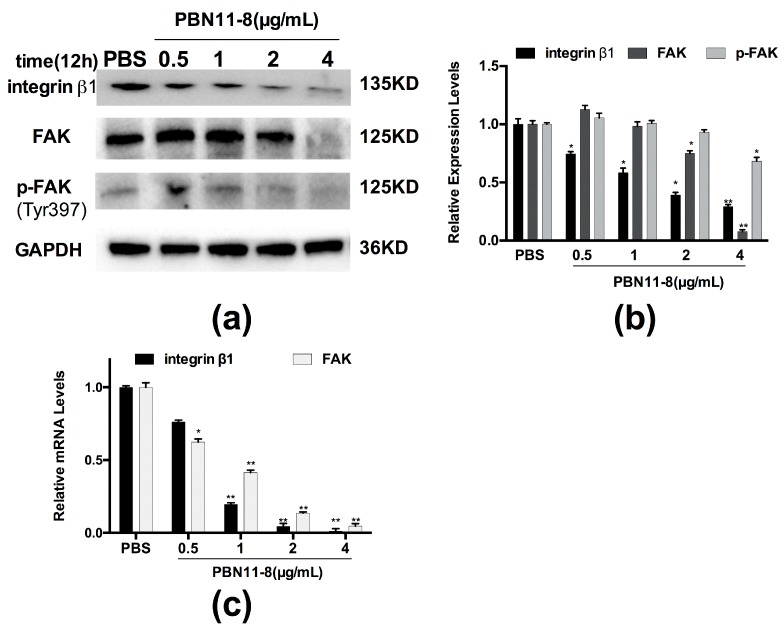
PBN11-8 inhibits the activation of FAK in BEL-7402 cells. (**a**) BEL-7402 cells were treated with (and without) 0.5, 1, 2, and 4.0 μg/mL PBN11-8. After incubation for 12 h, cells were collected and the cell protein was isolated for Western blot analysis of integrin β1, focal adhesion kinase (FAK), and p-FAK. Cells treated with PBS were used as negative control and the expression of GAPDH was used as an internal reference. (**b**) The quantitative evaluation of each lane of BEL-7402 cells in the Western blot was measured by Image J (National Institutes of Health, Bethesda, MD, USA) and normalized to untreated cells. (**c**) BEL-7402 cells were treated with (and without) 0.5, 1, 2, and 4.0 μg/mL PBN11-8. After incubation for 12 h, cells were collected and the total RNA was isolated. qRT-PCR analysis of integrin β1 and FAK was performed as described in Materials and Methods section. Cells treated with PBS were used as negative control and the expression of GAPDH was used as an internal reference. Data represent the mean ± SD of three independent experiments. * *p* < 0.05 vs. control; and ** *p* < 0.01 vs. control.

Studies have shown that integrin β1 is closely associated with adhesion, migration, and invasion and the biological function of integrin β1 is known to be partially mediated by the activation of FAK [24]. The expression levels of FAK are low in normal cells but FAK is highly expressed in primary and metastatic tumors. Increased expression of FAK in hepatocarcinoma cells is not only related to the degree of progression, but also related to the survival time of postoperative patients [25,26] Thus, FAK can be used as a marker for predicting malignancy and represents a potential target for future drug development. Western blot analysis was used to detect the expression of integrin β1 in BEL-7402 (Figure 5) to evaluate the possible molecular mechanism underlying the migration and invasion effects of PBN11-8. Our results showed that, compared with the control group, the expression of FAK and p-FAK was decreased in a concentration-dependent relationship in BEL-7402 cells (Figure 5a,b) treated with PBN11-8. The integrin β1 expression was also downregulated in BEL-7402 cells. qRT-PCR also showed that the relative mRNA levels of integrin and FAK was decreased in a concentration-dependent manner in BEL-7402 cells (Figure 5c) treated with PBN11-8. These results suggested that decreased expression of p-FAK is a necessary event in PBN11-8 induced cell growth inhibition and may contribute to its inhibitory activity of cell migration and invasion.

### 3.7. PBN11-8 Inhibited the Activation of ERK in BEL-7402 Cells

**Figure 6:**
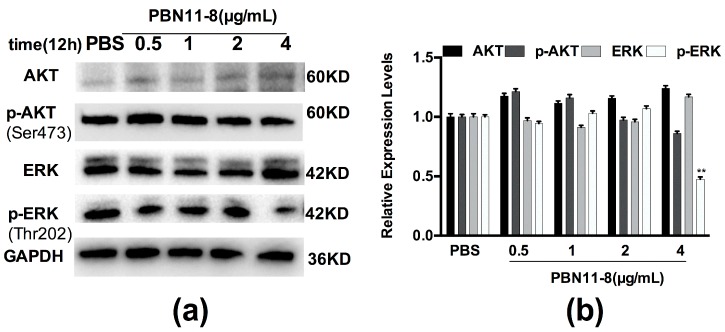
PBN11-8 suppressed extracellular regulated protein kinases (ERK) in BEL-7402 cells. (**a**) BEL-7402 cells were treated with (and without) 0.5, 1, 2, and 4.0 μg/mL PBN11-8. After incubation for 12 h, cells were collected and the cell protein was isolated for Western blot analysis of AKT, p-AKT, ERK, and p-ERK to be performed. Cells treated with PBS were used as negative control and the expression of GAPDH was used as an internal reference. (**b**) The quantitative evaluation of each lane of BEL-7402 cells in the Western blot was measured by Image J and normalized to untreated cells. Data represent the mean ± SD of three independent experiments. ** *p* < 0.01 vs. control.

The expression of AKT and ERK were investigated to further confirm whether the key target protein of FAK downstream signal pathway was altered in cells treated with PBN11-8. The results showed that the expression of AKT and ERK protein were unaltered in BEL-7402 cells treated with 0.5, 1.0, 2.0, or 4.0 µg/mL PBN11-8 for 12 h. P-AKT protein were decreased slightly in BEL-7402 cells treated with 4.0 µg/mL PBN11-8. However, the expression of p-ERK was significantly decreased in BEL-7402 cells treated with 4.0 µg/mL PBN11-8 (Figure 6a,b). These results indicate that PBN11-8 inhibits the activation of ERK.

### 3.8. Expression of MMP-2 and MMP-9 Was Reduced by PBN11-8 in BEL-7402 Cells

**Figure 7:**
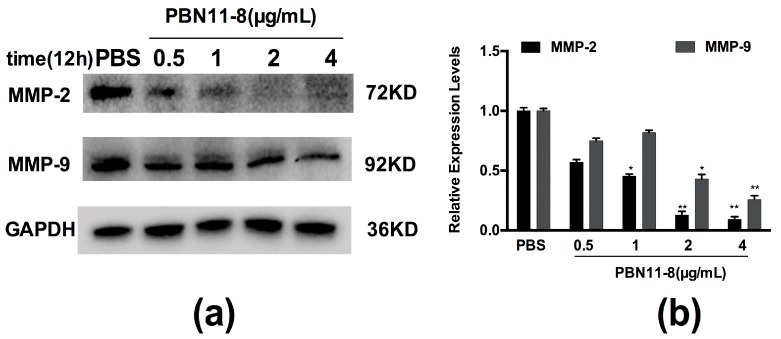
PBN11-8 affects MMP-2 and MMP-9 in BEL-7402 cells. (**a**) BEL-7402 cells were treated with (and without) 0.5, 1, 2, and 4.0 μg/mL PBN11-8. After incubation for 12 h, cells were collected and the cell protein was isolated for Western blot analysis of MMP-2 and MMP-9. Cells treated with PBS were used as negative control and the expression of GAPDH was used as an internal reference. The quantitative evaluation of each lane of (**b**) BEL-7402 cells was measured by Image J and normalized to untreated cells. Data represent the mean ± SD of three independent experiments. * *p* < 0.05 vs. control; and ** *p* < 0.01 vs. control.

It has been documented that Matrix metalloproteinases (MMPs) play a crucial role in tumor invasion and metastasis by degradation of the extracellular matrix, and high level expression of MMP-2 and -9 is correlated with an aggressive cancers [6]. We examined the effects of PBN11-8 on MMP-2 and MMP-9 expression in BEL-7402 cells. PBN11-8 had an inhibitory effect on MMP-2 and MMP-9 in BEL-7402 cells (Figure 7a,b) in a significant dose-dependent relationship. These results indicated that the downregulation of MMPs is also an important event in PBN11-8 induced inhibition on cell migration and invasion.

## 4. Discussion

Bioactive substances with antitumor activity from marine bacteria have recently been widely reported [15,18]. However, only a few bioactive polypeptides from marine microorganisms have been studied. In the current study, we employed complicated purification steps, including ammonium sulfate precipitation, Q anion exchange chromatography, and high performance liquid chromatography to obtain a polypeptide with cancer cells cytotoxicity from marine *Bacillus*. Sequence analysis revealed that the polypeptide, PBN11-8, belongs to a member of the peptidase M84 family; this is the first reported member of this family to display antitumor activity.

PBN11-8 has metalloproteinase activity, resulting from an active site and Met-turn motif known as MprBi [27,28]. It has been established that FAK is able to interact with integrin, PI3K [29], and STAT1 [30]. Downregulation of FAK plays an important role in PBN11-8 induced cell death. PBN11-8 prevents the adhesion, migration, and invasion of BEL-7402 cells as analyzed in morphological assessment, Scratch-wound assay and Transwell assay. Migration and invasion is tightly regulated by a large number of receptors, such as integrin, FAK, and MMPs. The integrin family of proteins can interact with membrane molecules, transferring the extracellular signal into the cytosol, and thus affect the expression of effector genes and the reorganization of the cytoskeleton. Moreover, integrin β1 cytoplasmic domains and other adaptor proteins, such as FAK, associated with the cell membrane interact with integrin, forming a focal adhesion complex. FAK also plays a critical role in tumor progression and metastasis through its regulation of cancer cell migration, invasion, epithelial to mesenchymal transition, and angiogenesis. These processes are involved with both cancer cells and their microenvironment [31]. In the present study, we found that PBN11-8 could reduce the expression of integrin, FAK, and p-FAK in protein levels. qRT-PCR results also showed that the mRNA level of integrin and FAK was decreased in BEL-7402 cells treated with the anticancer protein. These results suggest that PBN11-8 is capable of inhibiting the activation of FAK, and the activation of FAK plays a critical role in PBN11-8 induced cell growth inhibition, as well as cell invasion and migration.

The ERK signaling pathway is involved in multiple processes such as cell proliferation, invasion, angiogenesis and motility, which can also be regulated by the integrin/FAK network. Because of its multiple roles in the acquisition of a complex malignant phenotype, inhibition of the ERK pathway is expected to result in not only an anti-proliferative effect but also in anti-metastatic effects in tumor cells [32]. Nobiletin inhibits human osteosarcoma U2OS and HOS cells’ motility, migration and invasion by down-regulating MMP-2 and MMP-9 expressions via ERK pathways [33]. The important downstream genes, ERK, were also investigated in the present study to determine the effects of PBN11-8 inhibited FAK on downstream signaling and cell phenotypes. In our present study, we found that PBN11-8 is able to inhibit the ERK phosphorylation/activity. Further, the results showed that PBN11-8 inhibited the tumor cells through reducing their migration and invasion. The degradation of the ECM is mediated by proteases, such as MMPs. MMP-2 and MMP-9 are commonly expressed in many malignant tumors. It is well established that MMPs are able to degrade the ECM and basement membrane, promoting cancer metastasis, and MMPs are considered as important anticancer drug targets [34,35]. Both MMP-2 and MMP-9 are markers associated with the tumor invasion and metastasis [13]. In the present study, we reveal that PBN11-8 inhibits cell migration by down regulation of the MMP-2 and MMP-9 in BEL-7402 cells. The study provides primary evidence that the anticancer protein has potential to be developed as a novel anticancer agent used to treat cancer metastasis.

## 5. Conclusions

In summary, our present study reveals that PBN11-8 is able to significantly inhibit the growth of cancer cells, and inhibit adhesion, migration, and invasion by disturbing the integrin/FAK/ERK signal transduction and reducing MMP-2 and MMP-9 in BEL-7402 cells, and that the inhibitory effect of PBN11-8 on cells is highly associated with FAK expression. Our study suggests that PBN11-8 has the potential of being developed as a candidate drug for targeting FAK.
