# Correction: Zheng, L., et al. PBN11-8, a Cytotoxic Polypeptide Purified from Marine *Bacillus*, Suppresses Invasion and Migration of Human Hepatocellular Carcinoma Cells by Targeting Focal Adhesion Kinase Pathways. *Polymers* 2018, *10*, 1043

**DOI:** 10.3390/polym13010166

**Published:** 2021-01-05

**Authors:** Lanhong Zheng, Xiangjie Zhu, Kangli Yang, Meihong Zhu, Ammad Ahmad Farooqi, Daole Kang, Mi Sun, Yixin Xu, Xiukun Lin, Yingang Feng, Fangfang Liang, Fuming Zhang, Robert J. Linhardt

**Affiliations:** 1 School of Pharmacy, Shanghai University of Medicine & Health Sciences, Shanghai 201318, China; zhenglanhong@126.com (L.Z.); xuyx@sumhs.edu.cn (Y.X.); 2 Key Laboratory of Sustainable Development of Polar Fishery, Ministry of Agriculture, Yellow Sea Fisheries Research Institute, Chinese Academy of Fishery Sciences, Qingdao 266071, China; zhuxiangjie1204@163.com (X.Z.); hnysykl@163.com (K.Y.); zhumhlv@163.com (M.Z.); kdlqust@126.com (D.K.); sunmi@ysfri.ac.cn (M.S.); 3 Shanghai Ocean University, Shanghai 201306, China; 4 Institute of Biomedical and Genetic Engineering (IBGE), Islamabad 44000, Pakistan; ammadfarooqi@rlmclahore.com; 5 Department of Pharmacology, Southwest Medical University, Luzhou 646000, Sichuan, China; 6 Shandong Provincial Key Laboratory of Energy Genetics and Qingdao Engineering Laboratory of Single Cell Oil, Institute of Bioenergy and Bioprocess Technology, Chinese Academy of Sciences, 189 Songling Road, Qingdao 266101, China; fengyg@qibebt.ac.cn; 7 Departments of Chemistry and Chemical Biology, Chemical and Biological Engineering, Biology and Biomedical Engineering, Center for Biotechnology and Interdisciplinary Studies, Rensselaer Polytechnic Institute, Troy, NY 12180, USA; liangfangfang12@163.com (F.L.); zhangf2@rpi.edu (F.Z.); linhar@rpi.edu (R.J.L.)

The authors wish to make a change to the published paper [1]. In the original manuscript, the subfigures (0 h and 12 h) were repeated by mistake in Figure 3d. The corrected Figure 3 is presented below:

The authors apologize for any inconvenience caused and the change does not affect the scientific results. The manuscript will be updated, and the original will remain online on the article webpage at https://www.mdpi.com/2073-4360/10/9/1043.

## Figures and Tables

**Figure 3:**
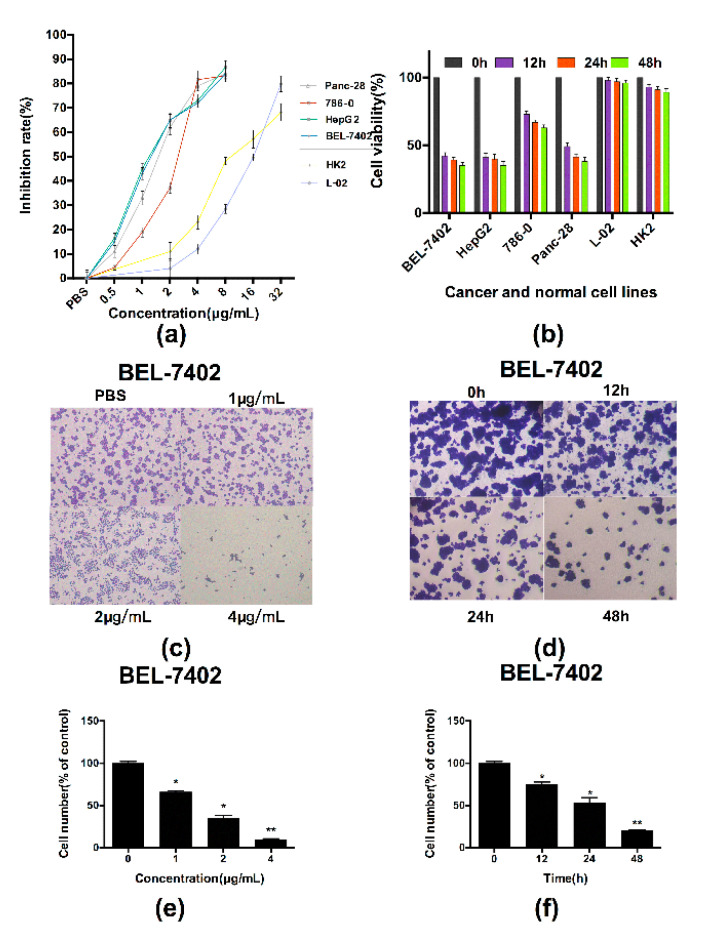
PBN11-8 displays potent cytotoxicity to cancer cells. (**a**) Cells were treated with certain concentrations of PBN11-8. The cell inhibitory rate was determined by MTT assay as described in the experimental section. The IC_50_ values were 1.56, 1.80, 1.57 and 1.73 µg/mL for BEL-7402, 786-0, HepG2 and Panc-28 cells, respectively. The IC_50_ values were 11.79 and 14.72 µg/mL for HK2 and L02 cells, respectively. (**b**) Cells were cultured in a 96-well plate and treated with 4 µg/mL PBN11-8 for each cell line for 0, 12, 24, and 48 h to study the time dependent analysis. Cell viability was analyzed by MTT assay. (**c**) The results of the crystal violet adhesion assay in BEL-7402 cells induced by 1, 2, 4 µg/mL of PBN11-8 for 12 h. (**d**) The results of the crystal violet adhesion assay in BEL-7402 cells induced by 2 µg/mL of PBN11-8 for 12, 24 and 48 h. (**e**,**f**) The quantitative evaluations of the crystal violet adhesion assay. The data represent the mean ± SD of three independent experiments. * *p* < 0.05 vs. control; and ** *p* < 0.01 vs. control.
